# INDOOR TEMPERATURES AND MENTAL HEALTH IN COMMUNITY-DWELLING OLDER ADULTS: A SYSTEMATIC REVIEW

**DOI:** 10.1093/geroni/igae098.4007

**Published:** 2024-12-31

**Authors:** Min Kyung Park, Dahye Hong, Seolah Yoon, Yubeen Kim, Minsub Kim, Bada Kang

**Affiliations:** Yonsei University College of Nursing, Seoul, Republic of Korea; Yonsei University, Seoul, Republic of Korea; Yonsei University, Seoul, Republic of Korea; Yonsei University College of Nursing, Seoul, Republic of Korea; Yonsei University College of Nursing, Seoul, Republic of Korea; Yonsei University College of Nursing, Seoul, Republic of Korea

## Abstract

The relationship between indoor temperature and mental health is becoming increasingly important as climate change leads to extreme temperature fluctuations. Older adults are particularly vulnerable to indoor temperature changes because of their diminished ability to regulate body temperature and because they spend a significant amount of time indoors. This systematic review examined the relationship between indoor temperature and mental health outcomes among community-dwelling older adults. A comprehensive search of seven electronic databases (MEDLINE, Embase, Cochrane Library, CINAHL, PsycINFO, Google Scholar, and PROQUEST) was conducted on April 4, 2024. Of the 2,328 identified studies, 15 met the inclusion criteria. Mental health outcomes associated with indoor temperature were categorized into four groups: sleep problems such as insomnia and sleep disturbance; emotional problems such as emotional distress and negative mood; social interaction problems such as social exclusion and low social participation; and other mental health issues such as anxiety, agitation, and annoyance. Sleep problems were the most frequently reported mental health outcome related to indoor temperature (n=9). Older adults living in substandard housing conditions, those with economic difficulties, and those residing in urban areas were vulnerable to exposure to hot or cold indoor temperatures because of housing-related risks such as low energy efficiency, inadequate heating or cooling, and limited access to green spaces. These findings highlight the need for evidence-based guidelines to improve mental health by ensuring appropriate indoor temperatures in the community. Improving housing conditions through policy support could be an effective strategy for enhancing the mental health of community-dwelling older adults.

